# Novel Feature Modelling the Prediction and Detection of sEMG Muscle Fatigue towards an Automated Wearable System

**DOI:** 10.3390/s100504838

**Published:** 2010-05-12

**Authors:** Mohamed R. Al-Mulla, Francisco Sepulveda

**Affiliations:** School of Computer Science and Electronic Engineering, University of Essex-Wivenhoe Park, Colchester CO4 3SQ, UK; E-Mail: fsepulv@essex.ac.uk

**Keywords:** sEMG feature extraction, muscle fatigue, 1D spectro, 1D spectro_std, Transition-to-Fatigue, peripheral fatigue

## Abstract

Surface Electromyography (sEMG) activity of the biceps muscle was recorded from ten subjects performing isometric contraction until fatigue. A novel feature (1D spectro_std) was used to extract the feature that modeled three classes of fatigue, which enabled the prediction and detection of fatigue. Initial results of class separation were encouraging, discriminating between the three classes of fatigue, a longitudinal classification on Non-Fatigue and Transition-to-Fatigue shows 81.58% correct classification with accuracy 0.74 of correct predictions while the longitudinal classification on Transition-to-Fatigue and Fatigue showed lower average correct classification of 66.51% with a positive classification accuracy 0.73 of correct prediction. Comparison of the 1D spectro_std with other sEMG fatigue features on the same dataset show a significant improvement in classification, where results show a significant 20.58% (p < 0.01) improvement when using the 1D spectro_std to classify Non-Fatigue and Transition-to-Fatigue. In classifying Transition-to-Fatigue and Fatigue results also show a significant improvement over the other features giving 8.14% (p < 0.05) on average of all compared features.

## 1. Introduction

Muscle fatigue is a reduction of the ability to contract and exert force. Generally, localized muscle fatigue occurs after a prolonged, relatively strong muscle activity, when a muscle or a group of muscles are fatigued. Due to the variability of the muscle characteristics from person to person there is no simple function of muscle load and timing that defines a precise muscle fatigue threshold. When muscle fatigue is not detected soon enough, it can often inflict injuries, causing not only pain to the subject but often a financial burden as well, especially for professional athletes [1]. It is also important to detect and classify muscle fatigue for use in human-computer interaction [2]. If fatigue occurs it causes degradation in the sEMG pattern recognition. To overcome this issue a detection or prediction of sEMG muscle fatigue must take place followed by adaption to the current rate of muscle fatigue enabling the pattern recognition process to be more robust. Myoelectric manifestations of muscle fatigue are perceived as an objective mean for the analysis of muscle fatigue as it disregards subjective motivators, and compared to mechanical measures it provides early indicators of fatigue. These manifestations refer to changes in signal frequency and amplitude and in the muscle conduction velocity (CV), while the mechanical factors relate to a loss in the exerted force [3].

Studies on muscle fatigue during isometric contraction have established typical sEMG readings when conducted in controlled settings. Changes in sEMG amplitude and centre frequency were studied by Petrofsky *et al.* [4]. The authors found a decrease in the centre frequency of the spectrogram of all the muscle groups. Research in this field also shows that a development in muscle fatigue correlates to changes in amplitude and median frequency (MDF) [5]. Kumar *et al*. have discussed the effectiveness of using the wavelet transform to decompose the signal to measure its power to identify muscle fatigue on EMG signals, which can be applied in an automated process for identifying fatigue [6].

A variety of other parameters have been used to investigate sEMG signals. Sung *et al.* [7] argues that entropy reveals part of the sEMG signals that are not included in the power spectrum, and it can be a useful tool in detecting muscle fatigue in gender differences. A fractal indicator is sensitive to the force level in isometric contractions, and is proved to be a good measure of muscle fatigue [8,9]. Gang *et al*. used a multifractal analysis to study the sEMG signals during static contractions [10]. They found that the spectrum increases and hence can be an indicator of fatigue; this method had higher sensitivity compared to the median frequency. Recurrence quantification analysis is highly effective in detecting changes in the sEMG signal and in comparison to the frequency domain analysis of the signal in non-isometric contraction; it is almost an equivalent mean [11]. Recent research by Morana *et al*. also used recurrence quantification analysis in their study of muscle fatigue and stated that this method can detect the peripheral of muscle fatigue [12]. Kim *et al.* studied the sensitivity of the first autogressive model to measure fatigue in the trunk muscle and concluded that this model can asses fatigue in static exercises, being a sensitive measure that can detect fatigue at low force levels [13]. Studies by Asghari Oskoei *et al*. stated that a significant decline in the signals Instantaneous Median Frequency (IMF) is the manifestation of fatigue taking place [3].

Our group previously conducted a study aimed at classifying the three classes of fatigue (Non-Fatigue, Transition-to-Fatigue and Fatigue) using Genetic Programming (GP) and a primitive function set, where the evolved solution was able to classify the unseen sEMG signal to an average of 83.17% accuracy in ten different individuals [14]. In another research, we looked at the possibility of differentiating the three classes of muscle fatigue (Non-Fatigue, Transition-to-Fatigue and Fatigue) using nine different features [15]. That study concluded with a clear discrimination between the three classes giving an estimate of 81.18% average change between the three classes.

Many past studies of sEMG localized muscle fatigue have been conducted on only the extremes of the signal (Fatigue or Non-fatigue), discarding the transitional state. In one of our recent studies we developed a new feature 1D spectro that managed to detect the onset of Transition-to-Fatigue with correct classification of 85% enabling the prediction of fatigue [16]. In this study, however, we focused on developing and analyzing a novel feature that was built on 1D spectro that can correctly detect the onset of Transition-to-Fatigue class followed by the detection of a muscle fatigue class. Once that was achieved we measured the classification performance of this feature, that we named 1D spectro_std. This new feature is looking at all the three classes of fatigue, but is classifying only two of them at the time (Non-Fatigue with Transition-to-Fatigue, and Transition-to-Fatigue with Fatigue). The aim of developing this new feature is for its future implementation in a wearable device that can autonomously predict and detect muscle fatigue using only sEMG signal while requiring low processing power, and yet be powerful in its applications.

## 2. Experimental Section

In the first stage of this research an experimental study was conducted to record sEMG emanating from the biceps brachii muscle. The second stage involved labeling the sEMG signal into three classes by using the mechanical aspects of muscle fatigue, such as elbow angle and arm oscillation into a fuzzy classifier, followed by validating the output of the fuzzy classifier by a human expert. In the third stage we used two extracted features to generate a novel feature (1D spectro_std) that will predict the onset of Transition-to-Fatigue and detect the onset of fatigue. Finally we analyzed, compared and validated the novel feature.

### 2.1. sEMG Recording and Pre-Processing

The data were collected from ten athletic, healthy subjects (mean age 27.5 ± 3.6 yr), all non-smokers. The ten participants were willing to reach physical fatigue state but not a psychological one (self-perceived fatigue). The participants were seated on a preacher curl machine to ensure stability and biceps isolation.

Steps in the test bed set up:

- sEMG electrodes were placed on the participant’s biceps brachii lower belly avoiding the estimated innervation zone (biceps belly) and toward the distal tendon to acquire sEMG reading. This electrode location was chosen as it facilitates an autonomous fatigue prediction/detection with reliable/repeatable data acquisition in realistic conditions. In addition it is a simple procedure for placing the electrodes when used by non-experts.
- Goniometer was placed on the side of the arm to measure the elbow angle and arm oscillation.
- The participants had a display placed in front of them, which indicated the elbow angle.
- The weight was handed to the participant at 90° elbow angle.
- Participants were asked to maintain the 90° angle.
- Participants stopped when they reached total biceps fatigue.
- All participants carried out the isometric exercises with 40% Maximum Voluntary Contraction (MVC).

The test bed set up for one of the conducted trials is shown in the picture below:

**Figure:**
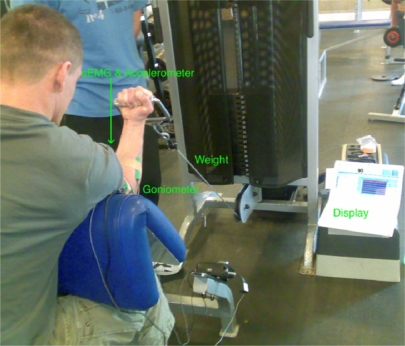

The myoelectric signal was recorded using two channels, Double Differential (DD) electrodes (Biometrics Ltd.), with A/D conversion at 2,000 samples/s. The sEMG signals acquired from the experiment were only filtered with a dual pass Butterworth filter, with the fifth order passband being between 1 and 500 Hz. The Goniometer readings were also recorded simultaneously. The reading of the Goniometer was then correlated with the sEMG signal to ensure that fatigue resides within the sEMG. The Goniometer provided a reliable mechanical indication on the development of fatigue. Classical biceps muscle fatigue for the clinically healthy individuals usually manifest itself by small oscillation or vibrations followed by a difficulty in maintain a task [14–16], in our case the 90° elbow angle. For each of the ten participants three trials were carried out, providing 30 trials in total. There was a resting period of one week between each of the three trials ensuring full recovery of the biceps brachii.

### 2.2. sEMG Segment Labeling

In labeling the sEMG only the kinematic aspects, such as a drop in the elbow angle and oscillation (*i.e.*, standard deviation of the elbow angle), were considered, as they are the most reliable indicators in healthy individuals when assessing muscle fatigue. The use of the kinetic variables defines the boundaries (Non-Fatigue, Transition-to-Fatigue and Fatigue) of the sEMG signal, providing the basis for training the sEMG classifier. In our case the Goniometer signal contains (elbow angle and oscillation) the physical manifestation of fatigue (Non-Fatigue, Transition-to-Fatigue and Fatigue).

**Figure 1.**
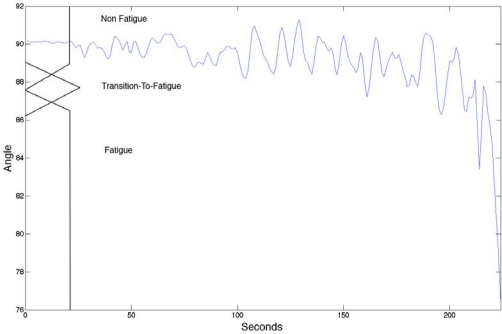
The fuzzy set input for the angular position of the elbow.

**Figure 2.**
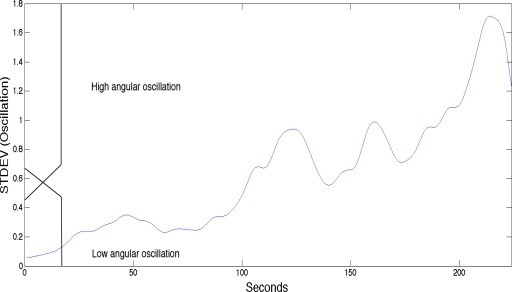
The fuzzy set input for the angular oscillation.

In this study we used both a fuzzy classifier to automate the labeling and a human expert to verify the outcome of the fuzzy classifier. The two main criteria in labeling the sEMG signal are described below using fuzzy logic terms. The fuzzy logic had two inputs:

- Figure 1 indicates the fuzzy set input for the elbow angle provided by the Goniometer (0 to 180 degrees): Angles above 89 indicates Non-Fatigue, angles below below 86.5° indicates Fatigue and any angle between 86.5° to 89° boundaries are considered Transition-to-Fatigue. The figure also has a superimposed illustration of a single Goniometer trial signal giving an example of how the fuzzy classifier is finding the boundaries to enable the labeling of the sEMG signal.
- Figure 2 indicates the fuzzy set input for the arm oscillations, which was also provided by the Goniometer: An increase in the standard deviation of the Goniometer signals (calculation of the standard deviation was implemented using four second non-overlapping window of the Goniometer signal then resample to match the original signal size) indicates either low angular oscillation or high angular oscillation. Further examination of Figure 2, with the superimposed standard deviation signal of the of the Goniometer signals, reveals that at around 110 seconds (in this particular signal), which resides at 0.6 standard deviations, indicates transition from Non-Fatigue to Transition-to-Fatigue state [14,15].

In the experiments, the subjects were instructed to try to maintain a Goniometer angle of 90° until complete fatigue was reached.

For the fuzzy classifiers used in this study, only a single label was chosen as the final output. Table 1 below defines the rule base; the rule with the greatest firing strength was selected.

The above fuzzy classifier inputs, when used in conjunction, were found to assist in finding the boundaries of the classes. Both inputs were used to define a 6 rule type-1 fuzzy classifier; using both triangular and trapezoidal antecedents and product inference.

### 2.3. Feature Extraction

This study is looking at three classes of sEMG (Non-Fatigue, and Transition-to-Fatigue and Fatigue), which relate to the status of the muscle and will produce distinct features. The features were extracted by using other features that have been used in previous studies to extract fatigue content in EMG [17]. The feature extraction methods described in the following subsections are the building blocks for constructing the novel sEMG feature developed in this study. It is important to note that the sEMG signal was split into one-second intervals during the feature extraction processes.

#### 2.3.1. Instantaneous Median Frequency

The spectral frequency can be redefined to contain time dependence of the signals frequency content [18], and time dependence can be referred to as the instantaneous frequency. The instantaneous median frequency was introduced by Roy *et al*. who depicted the following formula where t is time, P is Power spectrum density function, w is the window size and d is the depth of the signal [19].

(1) $$\int_{0}^{\mathrm{IMDF}(t)}P(t,w)dw=\int_{\mathrm{IMDF}(t)}^{\infty }P(t,w)dw$$

#### 2.3.2. Total Band Power of sEMG

The total band power of the sEMG was estimated using Welch’s method. This method was used in several sEMG fatigue analysis and proved to be useful in quantifying the power of the EMG signals [20].

#### 2.3.3. Novel Feature (1D spectro_std)

**Figure 3.**
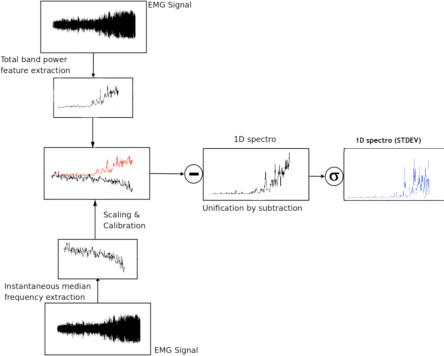
An illustration on constructing the 1D spectro_std feature.

As mentioned above, the 1D spectro_std was developed in this study to assist in the prediction and detection of muscle fatigue and was built on a previous study that predicted the onset of Transition-to-Fatigue class using the feature 1D Spectro [16]. In that study the instantaneous median frequency and the total band power are unified. The novel feature in this research was produced by using the standard deviation of the unified signal which we named 1D spectro_std. The name of this feature was inspired by the spectrogram. The spectrogram shows the spectral density over time usually using a two- dimensional image, while the 1D spectro reduces the dimensionality to one. This is accomplished when the two feature signals are unified (total band power and instantaneous median frequency) by subtraction. Due to the simplicity and powerful analysis capabilities, the 1D spectro_std can be easily embedded in a mobile wearable muscle fatigue system while retaining exceptional classification performance. Figure 3 shows a graphical representation of how the updated 1D spectro forms the 1D spectro_std. The latter is used in this study to detect the onset of Transition-to-Fatigue and the onset of Fatigue.

**Figure 4.**
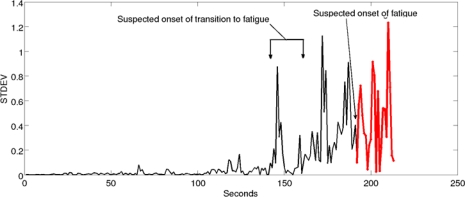
Illustration of the three classes of fatigue for one of the trials.

The 1D spectro_std is light on the resources, however it is still reliable in real-time prediction and detection of fatigue. It was established in this research that once the onset of Transition-to-Fatigue has taken place the output of the 1D spectro_std value will increase, creating large spikes within the signal. This is contrary to when fatigue onset is reached, where the 1D spectro_std values will start to drop drastically, as shown in Figure 4. This phenomenon directly correlates to the fuzzy classifier that was used to label the signal which was based on the mechanical aspects using the Goniometer (drop in elbow angle and arm oscillations). Figure 4 illustrates these findings when extracting the 1D spectro_std from the sEMG for one of the trials that contain Non-Fatigue, Transition-to-Fatigue and Fatigue segments. (See Figure 1, Figure 2 showing the Goniometer feedback while Figure 4 shows the outcome of the feature extracted from the sEMG.)

### 2.4. Validation/Classification

In order to compare and validate the new features with other features, a linear discriminant analysis (LDA) was used. The following linear transformation describes the classification where the LDA maps the data (feature vector) x:

(2) $$y=W^{T}x+w_{0}$$

where W and w0 are determined by maximizing the ratio of between-class variance to within-class variance to guarantee maximal separability [21].

The classification is described below (In this study only two classes were used at a time, e.g., Non-Fatigue *vs.* Transition-to-Fatigue and Transition-to-Fatigue *vs.* Fatigue):

(3) $$\begin{array}{c} X\in \{\begin{array}{ll} \mathrm{Non}–\mathrm{Fatigue}, & \mathrm{if}\;y>0,\\ \mathrm{Transition}–\mathrm{To}–\mathrm{Fatigue}, & \mathrm{if}\;y<0. \end{array}\\ X\in \{\begin{array}{ll} \mathrm{Transition}–\mathrm{To}–\mathrm{Fatigue}, & \mathrm{if}\;y>0,\\ \mathrm{Fatigue}, & \mathrm{if}\;y<0. \end{array} \end{array}$$

In this study we have compared the novel feature 1D spectro_std classification performance with four other features, two of which (instantaneous median frequency and total band power) are described in Section 2.3. The latter two are described in the subsections below.

The classification performance of the novel feature 1D spectro_std was tested using longitudinal classification. When measuring the classification performance of all the compared features using the same dataset, the standard deviation was computed for each of the features with a window of three second to enable a similar criteria to the 1D spectro_std, hence results of these compared features can be correctly compared to the 1D spectro_std.

#### 2.4.1. New Spectral Index (FI2–FI5)

Dimitrov *et al*. suggested that a special parameter would give a much higher sensitivity for both dynamic and isometric contractions. The parameter used the fast Fourier transform to calculate ratios between different spectral moments measured over the power spectral density [22].

(4) $$\mathrm{FInsmX}=\frac{\int_{f1}^{f2}f^{-1}PS(f).df}{\int_{f1}^{f2}f^{X}PS(f).df}$$

where X is the moment and PS(f) is the EMG power spectrum calculated using

Fourier transform and f1 = 8 Hz and f2 = 500 Hz.

Only the FI2 spectral index was included in this study and compared in the final result as it gave the highest classification measure out of the other spectral indices (FI3–FI5).

#### 2.4.2. Wavelet Decomposition

In wavelet transforms (WT) there is a range of standard ‘mother’ wavelet functions to be used as basis for the decomposition of a signal, e.g., Symmlet, Coiflet, Haar, Morlet, Daubechies and Mexican Hat [6]. Some of the mother wavelets are more suited for a specific application and signal type, although there is no definite rule for the selection of a wavelet basis function. To select the appropriate wavelet the properties of the wavelet function and the characteristic of the signal needs to be analyzed and matched. However, some of the wavelets have somewhat established guidelines, e.g., Db4 is said to be suited for signals using feature extractions and linear approximation with more than four samples, while Db6 is used for a signal approximated by a quadratic function over the support of six; coiflet6 is better suited for data compression results [23]. Kumar *et al*. stated in their research that wavelets can be used to find fatigue content [6].

We have decomposed all our subjects sEMG signals at scale 12 (scale 12 produced the best correct classification performance) with DB2, DB3, DB4, Haar, Sym4 and Sym5 wavelets, then labeled them with same procedure mentioned in Section 2.2. We found that the DB3 wavelet produced the best classification performance out of the other wavelets and hence was used in this study to compare with the 1D spectro_std.

#### 2.4.3. Confusion Matrix

To measure the true performance of the classification we used a confusion matrix based on a classification system that contains information about actual and prediction classifications. The table below shows the entries in the confusion matrix as defined by Kohavi and Provost [24].

where,

*a* is considered a number of seconds of *correct* predictions when the occurrence is *negative*
*b* is considered a number of seconds of *incorrect* predictions when the occurrence is *positive*
*c* is considered a number of seconds of *incorrect* predictions when the occurrence is *negative*
*d* is considered a number of seconds of *correct* predictions while the occurrence is *positive*

For this two-class matrix, as in our study, several functions were used to get the outputs. To calculate the *True Positive rate (TP)*, which is the proportion of positive cases identified correctly, the following equation was used:

(5) $$TP=\frac{d}{c+d}$$

When a number of negative cases were incorrectly identified as positive, we get the *False Positive rate (FP)*:

(6) $$FP=\frac{b}{a+b}$$

To find the *True Negative rate (TN)*, where the proportion of negatives cases that were classified correctly, we used:

(7) $$TN=\frac{a}{a+b}$$

The *False Negative rate* (*FN*) is defined as the number of positives cases incorrectly classified as negative, calculated by the equation:

(8) $$FN=\frac{c}{c+d}$$

With the following equation *Precision* (*P*) is calculated, which is a number of correct predictions while the prediction is positive:

(9) $$P=\frac{d}{b+d}$$

### 2.5. Measure of Class Separation within the Novel Feature Using Davies Bouldin Index (DBI)

Further analysis of the novel feature was carried out to measure how it affects the standard deviation of the signal. Davies Bouldin Index (DBI) was used to quantify the standard deviation.

The purpose of DBI is to decide cluster quality; it is a measure of the nearness of the clusters’ members to their centroids and the distance between clusters’ centroids [25]. It is preferable that the DBI decreases. The closer the index is to zero, the better is the separation of the classes.

DBI can be expressed as follows: C_CLi_ is the centroid of the *CL_i_^th^* cluster and *d^n^_CLi_* the *n*^th^ data member that belongs to the *CL*_i_^th^ cluster. In addition, the Euclidian distance between *d^n^_CLi_* *d* and *C_CLi_* is expressed by the function be *dis(d_CLi_* *, C_CLi_).* Furthermore, *K* is the total number of clusters. Finally, standard deviation is denoted as std(). Thus,

(10) $$DBI=\frac{\sum_{i=1}^{k}\mathrm{Std}\left[\mathrm{dis}\left(C_{\mathrm{CLi},}d_{\mathrm{CLi}}^{0}\right),\ldots ,\mathrm{dis}\left(C_{\mathrm{CLi},}d_{\mathrm{CLi}}^{n}\right)\right]}{\mathrm{dis}\left(C_{\mathrm{CL}0,}C_{\mathrm{CL}1},\ldots ,C_{\mathrm{CLi},}\right)}$$

## 3. Results and Discussion

**Figure 5.**
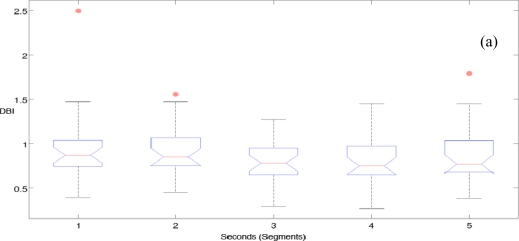

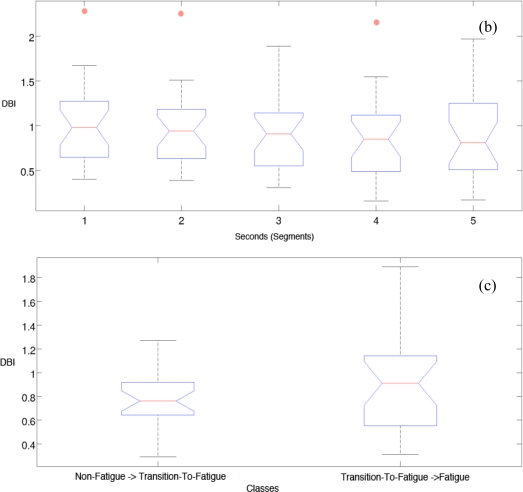
(a) Measuring class separation using DBI within 1 to 5 second segments of standard deviation for Non-Fatigue and Transition-to-Fatigue. (b) Measuring class separation using DBI within 1 to 5 second segments of standard deviation for Transition-to-Fatigue and Fatigue. (c) Comparison of Non-Fatigue to Transition-to-Fatigue DBI and Transition-to-Fatigue to Fatigue of the DBI when using three seconds segments.

Figure 5(a) shows the initial results of the DBI for Non-Fatigue and Transition-to-Fatigue. It can be seen that when we use more than one second the separation improves, thus improving the classification in later stages. It was chosen in this study to use a three second segment of standard deviation to improve performance while keeping the system sensitive when used in realistic scenarios.

Figure 5(b) displays the initial results of class separation using the DBI for Transition-to-Fatigue and Fatigue within 1 to 5 seconds of standard deviation segments. The three seconds segment improves the separation and was thus used in this study. Figure 5(c) shows only the three-second segments comparison between the classes (Non-Fatigue and Transition-to-Fatigue with Transition-to-Fatigue and Fatigue). It can be seen in Figure 5(c) that the separation of Non-Fatigue and Transition-to-Fatigue classes has on average a lower value when compared to the separation of Transition-to-Fatigue and Fatigue classes. Table 3 shows the classification performance of the 1D spectro_std against other features with the same datasets when classifying Non-Fatigue and Transition-to-Fatigue; giving an average of 81.58% correct classification, which outperformed the other four features. When compared to the feature that produced the highest classification, *i.e.*, wavelet decomposition DB3, 1D spectro_std shows an improvement of 13.49% (p < 0.01) in its correctness of classification. Moreover, 1D spectro_std shows a significant improvement when compared to all the averaged features giving 20.58% improvement (p < 0.01) in correctness of classification over all the other four compared features. These results can be compared to Table 5, which displays the correct classification performance of Transition-to-Fatigue and Fatigue. These results indicate that with an average performance of 66.59%, 1D spectro_std has a 5.64% improvement over the other feature that generated the highest percent of classification *i.e.* new spectral index (FI2). On average 1D spectro_std makes an improvement of 8.14% (p < 0.05) over the other four features for classifying Transition-to-Fatigue and Fatigue.

Tables 4 and 6 illustrate the confusion matrix results in seconds showing the true performance measure when using 1D spectro_std to classify the three classes (Non-Fatigue, Transition-to-Fatigue and Fatigue) averaged across the full set of subjects. By looking at Table 4, which is complementing Table 3 (Non-Fatigue and Transition-to-Fatigue) it can be seen that the accuracy of classification on the proportion of total number correct classification prediction is 0.74 where 1 is highest. This is a clear indication of validity of the results obtained in Table 3. It can also be seen from Table 4 that the rates of true positive and true negative reflect the results of the accuracy measure. Table 6 shows the confusion matrix for classifying Transition-to-Fatigue and Fatigue, complementing Table 5. Results exhibit an interesting behavior as the average of Table 5 was 66.59% correct classification. Table 6 shows somewhat positive results when looking at the true performance. When studying the accuracy in Table 6 it can be seen that it is very similar to the results obtained in Table 4, meanwhile the precision is showing a much lower value indicating that predicted true cases was lower than in Table 4. It can be also noted that in both confusion matrix Tables 4 and 6 the false negative is where the reduction in classification occurs and this can be explained by the nature of the signal.

The results in this study proved that the novel 1D spectro_std feature gives higher classification accuracy when compared to other features commonly used in sEMG muscle fatigue classification. This shows that the 1D spectro_std feature is an exceptional fatigue index that can be used in future research on sEMG signals to predict muscle fatigue by correctly classifying the Transition-to-Fatigue class.

The development of the new feature 1D spectro_std opens new doors for researchers to explore the idea of combining two or more features creating a composite feature that can increase the sensitivity in extracting muscle fatigue features, hence improving classification accuracy. Another positive point in our approach is that we did not discard any segments of the signal in the analysis process, contrary to most previous research that emphasize the peripheral of the signal [6]. The benefit of using the total length sEMG signal, is that it can empower any application that uses sEMG for its functionality while accounting and adapting for muscle fatigue. This is due to the nature of sEMG; in particular when peripheral fatigue sets in, the dynamics of the sEMG changes degrading the pattern recognition capabilities [2]. Recent research that investigates the peripherals of localized muscle fatigue [12,21] conforms to our findings. However, not much research has been conducted apart from our past research [14,15] to automate the process of predicting muscle fatigue by identifying and quantifying the Transition-to Fatigue (peripheral fatigue).

## 4. Conclusions

In this study we developed a novel feature, 1D spectro_std, with the intention to model localized muscle fatigue that can be used to predict fatigue by accurately detecting the Transition-to-Fatigue class and also detecting the Fatigue class. Results for detecting Transition-to-Fatigue gives an encouraging 81.58% correct classification with accuracy 0.74 of correct predictions. A comparison was made with other features classification capabilities on the same dataset to see where the 1D spectro_std stands. Results on this comparison show a significant improvement over the averaged classification of all four compared features to 20.58% (p < 0.01). Also in this study, by using the new feature 1D spectro_std, we managed to detect the onset of fatigue with an average correct classification of 66.59% with a positive classification accuracy 0.73 of correct predictions, and the comparison with the other four features showed a smaller but significant improvement of 8.14% (p < 0.05) for Transition-to-Fatigue and Fatigue on average over all four tested features.

The achievements reached by the 1D spectro_std to predict muscle fatigue by recognition of the Transition-to-Fatigue class opens the door for research to explore the generation of new composite features that would improve classification accuracy. We are currently exploring the technique of multi-feature fusion with very encouraging results, which we look forward to publishing shortly.

## Figures and Tables

**Table 1.** Rule base for signal labeling.

**Rules**	**IF Input 1 (Elbow Angle)**	**Input 2 (Angular oscillation)**	**THEN Output**
**1**	Non-Fatigue	Low	Non-Fatigue
**2**	Non-Fatigue	High	Transition-to-Fatigue
**3**	Transition-to-Fatigue	Low	Transition-to-Fatigue
**4**	Transition-to-Fatigue	High	Transition-to-Fatigue
**5**	Fatigue	Low	Fatigue
**6**	Fatigue	High	Fatigue

**Table 2.** Confusion matrix.

	**Predicted −**	**Predicted +**
**Actual −**	a	b
**Actual +**	c	d

**Table 3.** Percent correct classification for Non-Fatigue and Transition-to-Fatigue for the various features within subjects.

**Subject**	**1D spectro_std %**	**Instant. Median Freq. %**	**Total Band Power %**	**New Spectral Index (FI2) %**	**Wavelet (DB3) %**
1	82.1	53.2	73.31	62.95	74.43
2	86.27	50.32	62.4	51.75	61.33
3	82.55	54.71	59.49	55.55	62.61
4	75.69	49.79	55.94	63.02	54.82
5	84	55.77	67.53	53.35	75.93
6	86.84	51.52	59.14	57.38	71.46
7	75.88	60.36	57.67	63.11	74.39
8	80.71	51.4	72.22	60.46	72.24
9	88.93	55.47	64.88	65.09	61.36
10	72.86	55.71	62.94	52.44	72.33
**AVG**	81.58	53.83	63.55	58.51	68.09
**STDEV**	5.32	3.23	5.95	5.03	7.34

**Table 4.** Confusion matrix of 1D spectro_std for the classification of Non-Fatigue (NF) and Transition-to-Fatigue (TF) averaged across the full set of subjects.

**Average of all subjects in second**	**Predicted NF**	**Predicted TF**	
**Actual NF**	16	1	
**Actual TF**	8	11	
**True +**	0.58	**False +**	0.05
**True −**	0.95	**False −**	0.42
**Precision**	0.93	**Accuracy**	0.74

**Table 5.** Percent correct classification for Transition-to-Fatigue and Fatigue for the various features within subjects.

**Subject**	**1D spectro_std %**	**Instant. Median Freq. %**	**Total Band Power %**	**New Spectral Index (FI2) %**	**Wavelet (DB3) %**
1	53.75	53.35	54.01	58.53	58.33
2	80.86	57.5	45.41	58.82	61.62
3	79.13	54.17	54.56	54.37	73.25
4	35.62	59.8	57.84	53.92	50.88
5	70.09	48.4	59.29	64.15	67.19
6	57.88	56.94	50.69	79.17	59.42
7	85.88	55.44	60.55	68.77	68.08
8	69.89	51.24	53.9	64.82	52.77
9	64.06	65.66	61.74	60.63	57.16
10	68.75	58.33	66.41	46.28	54.27
**AVG**	66.59	56.08	56.44	60.95	60.30
**STDEV**	14.78	4.8	6.01	9.05	7.26

**Table 6.** Confusion matrix of 1D spectro_std for classification of Transition-to-Fatigue and Fatigue (NF = Non-Fatigue and TF = Transition-to-Fatigue) averaged across the full set of subjects.

**Average of all subjects in second**	**Predicted NF**	**Predicted TF**	
**Actual NF**	13	2	
**Actual TF**	3	4	
**True +**	0.62	**False +**	0.14
**True −**	0.86	**False −**	0.38
**Precision**	0.65	**Accuracy**	0.73
